# Recurrence of carcinoma showing thymus-like differentiation (CASTLE) involving the thyroid gland

**DOI:** 10.1186/s13044-021-00111-3

**Published:** 2021-08-16

**Authors:** N. V. Dang, L. X. Son, N. T. T. Hong, N. T. T. Nhung, N. T. Tung, L. V. Quang

**Affiliations:** 1 Department of Oncology, Hanoi Medical University, 01 Ton That Tung Street, Dong Da District, Hanoi, Vietnam; 2 Department of Head and Neck Radiation Oncology, Vietnam National Cancer Hospital, Hanoi, Vietnam; 3 Department of Radiation Physics, Vietnam National Cancer Hospital, Hanoi, Vietnam

**Keywords:** Thyroid cancer, CASTLE, Surgery, Radiotherapy

## Abstract

**Background:**

Carcinoma showing thymus-like differentiation (CASTLE) in the thyroid gland is a rare disease with generally a favorable prognosis. Treatment with surgery and adjuvant radiotherapy has been shown to improve local control and long-term survival rates. In this report, we present a case of a recurrent thyroid gland CASTLE and review the literature on the diagnosis and treatment of this disease.

**Case presentation:**

A 60-year-old woman, who was diagnosed with a CASTLE thyroid tumor in 2015, had a total thyroidectomy and was maintained on thyroid hormone replacement (levothyroxine). After 5 years, the patient had a recurrence, in an advanced stage unsuitable for surgery. As the patient declined to undergo radiotherapy, she was followed up without intervention and is currently stable after 15 months.

**Conclusions:**

CASTLE is a rare disease, diagnosed based on postoperative pathology and immunohistochemistry analysis, especially upon CD5 marker. In case of relapse, treatment options include surgery and radiotherapy; however conservative management without intervention is an acceptable alternative in some cases.

## Background

Carcinoma showing thymus-like differentiation (CASTLE) is a rare disease, which is often difficult to diagnose due to a lack of specific clinical characteristics. CASTLE tumor usually invades neighboring organs and metastasizes to local lymph nodes [1]. CASTLE tumor generally has a favorable prognosis, and there is no standard treatment although surgery with or without adjuvant radiotherapy is considered the first-line treatment. We report a case of CASTLE thyroid tumor which was treated with total thyroidectomy and lymph node dissection but relapsed 5 years after the surgery.

## Case presentation

**Fig. 1:**
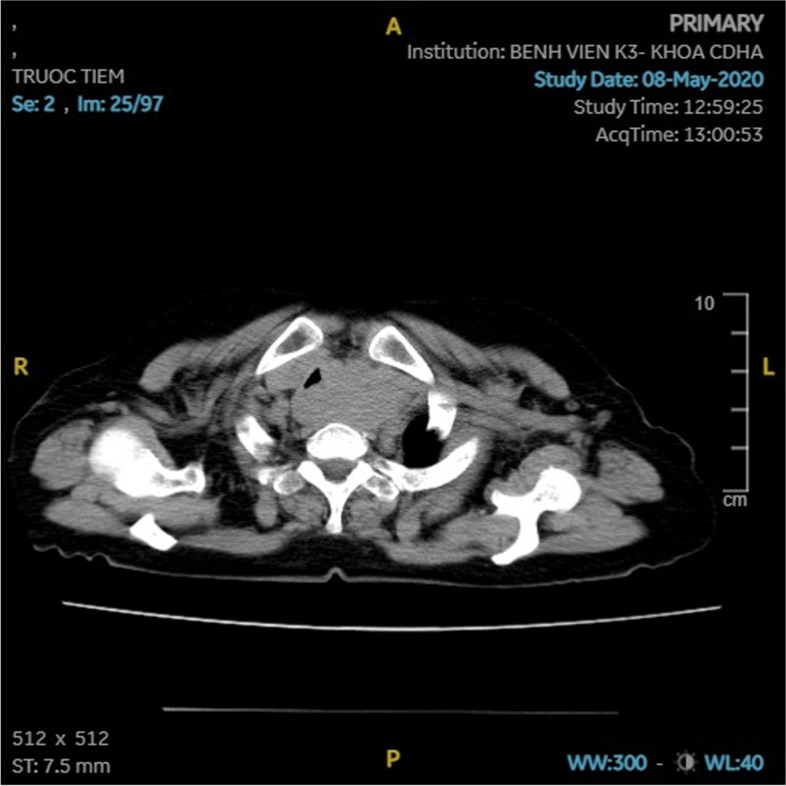
CT scan: The lesion was 16 × 54 mm in size, compressed the trachea

A 60-year-old woman, who was diagnosed with cancer of the thyroid gland in 2015, was treated with total thyroidectomy and lymph node dissection. Her postoperative pathology showed carcinoma showing thymus-like differentiation (CASTLE). She was discharged on Levothyroxine 100 mg/day. She did not attend her follow-up appointments. After 2 years, the patient found a lump in the left side of her neck. The tumor grew very slowly and did not cause any symptoms; hence, she did not seek medical advice. In May 2020, about 3 years after the appearance of her left neck tumor, the patient was admitted to our hospital with shortness of breath due to tracheal compression from the tumor. Clinical examination showed enlarged solid painless cervical lymph nodes at levels III and IV, stuck in a block size of 3 × 6 cm in diameter. There was no involvement of the hypopharynx, and her vocal cords were intact. CT scan of her neck revealed a 16 × 54 mm lesion compressing the trachea, and several enlarged left cervical lymph nodes with the size of about 25 mm (Fig. 1). The patient was offered a tracheostomy and tracheal stent before undertaking further investigations.

**Fig. 2:**
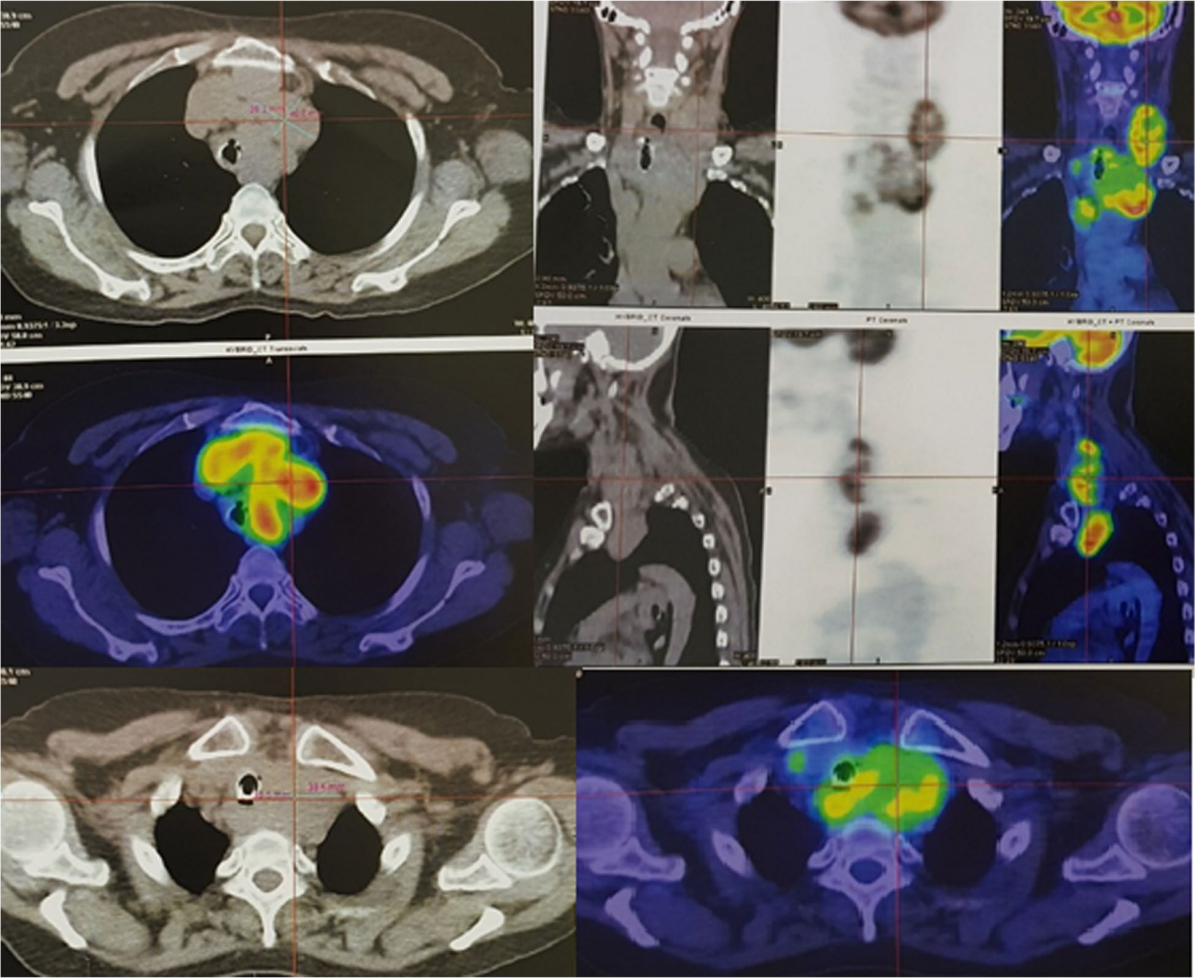
PET/CT: A 33 × 39x49 mm mass on the left side of the anterior mediastinum; multiple lymph nodes near the lower trachea, next to the superior vena cava, posterior

Her subsequent fluorine-18-labeled fluorodeoxyglucose positron emission tomography/computed tomography (FDG PET/CT) scan revealed previous total thyroidectomy, no evidence of recurrence in the surgical bed, enlarged left cervical lymph nodes at levels III and IV, and extranodal extension into sternocleidomastoid muscle measuring 29 × 43x59 mm, with the maximum standardized uptake value (SUV max) of 6.8. Her thymus measured 19 × 31x37mm, with increased metabolism of FDG (SUV max 3.6). There was a 33 × 39x49 mm mass on the left side of the anterior mediastinum, showing an undefined margin with neighboring organs in the trachea and the thorax (SUV max 6.0). It also showed multiple lymph nodes near the lower trachea, next to the superior vena cava, posterior thymus, and aortic loop; the largest node was 26 × 40 mm, with increased metabolism of FDG (SUV max 7.9). The other organs were unaffected. There was no metastasis in other organs (Fig. 2).

Thyroid function tests showed TSH: 0.00726 mIU/L[0,4-5], FT4: 22.34 pmol/L [12-22] and FT3: 4.0 pmol/L [1, 3-3, 1]. We reviewed the pathology of the current cervical lymph node specimen and that of the thyroid specimen from 5 years ago (2015). This showed that the thyroid tumor consisted of invasive cells which were poorly differentiated with squamous cell morphology, different from conventional thyroid carcinoma. The immunohistochemistry (IHC) analysis showed positive staining with CK5, CK7, p63, C-kit, CD5 and negative with TTF-1, Thyroglobulin, GATA3, ER, CD99, and NKX2.2, confirming CASTLE pathology in both the specimens.

The patient was diagnosed with a recurrence of the CASTLE thyroid gland in cervical lymph nodes. She was assessed by thoracic, and head and neck surgeons for the possibility of removing the maximum of recurrent tumors. However, it was considered a difficult, high-risk procedure; she and her family declined to go ahead with the surgery. Then, we offered the patient treatment with palliative radiotherapy. However, she and her family declined the treatment and got the patient discharged from the hospital. She was followed up without intervention and is currently stable 15 months after the recurrence.

## Discussion

CASTLE is a rare disease of unknown etiology, accounting for 0.1–0.15% of all thyroid gland cancer. It commonly occurs at around the age of 40 to 50 years and has a slight female predominance. It was first described by Miyauchi et al. in 1985 and named “thymus carcinoma in the thyroid” [2]. In 1991, Chan and Rosai classified this type of tumor into four groups: ectopic hamartomatous thymoma, ectopic cervical thymoma, spindle epithelial tumors with thymic-like differentiation (SETTLE), and carcinoma showing thymus-like elements (CASTLE) [3]. SETTLE and CASTLE show characteristics of malignant tumors, the other two pathologies are considered as benign tumors. SETTLE commonly occurs among the young, while CASTLE is more common at the ages of 50 [4, 5].

The histopathology and immunohistochemistry of the CASTLE tumor show feature similar to thymus carcinoma cancer and possibly due to initial origin in the thymus gland or branchial pouch. The immunohistochemical test shows characteristic CD5-positive staining in most cases and negative staining with thyroid gland markers such as thyroglobulin and calcitonin. Molecular analysis shows p63-positive on most tumors of thymus origin, but negative results are found on cystic carcinoma and poorly differentiated form in the thyroid gland [6].

CASTLE generally shows indolent growth and has a favorable prognosis. Most patients present with a painless, slowly growing mass in the neck. Some patients may also experience hoarseness or difficulty in swallowing due to tumor invasion in neighboring soft tissues and regional lymph nodes. Other symptoms include a dry cough and short of breath [7]. In our patient, after initial total thyroidectomy, the recurrence was associated with slowing growing cervical lymph nodes over 3 years resulting in tracheal compression. It has been reported that CASTLE can rarely metastasize to the brain, liver, and lungs [8].

**Fig. 3:**
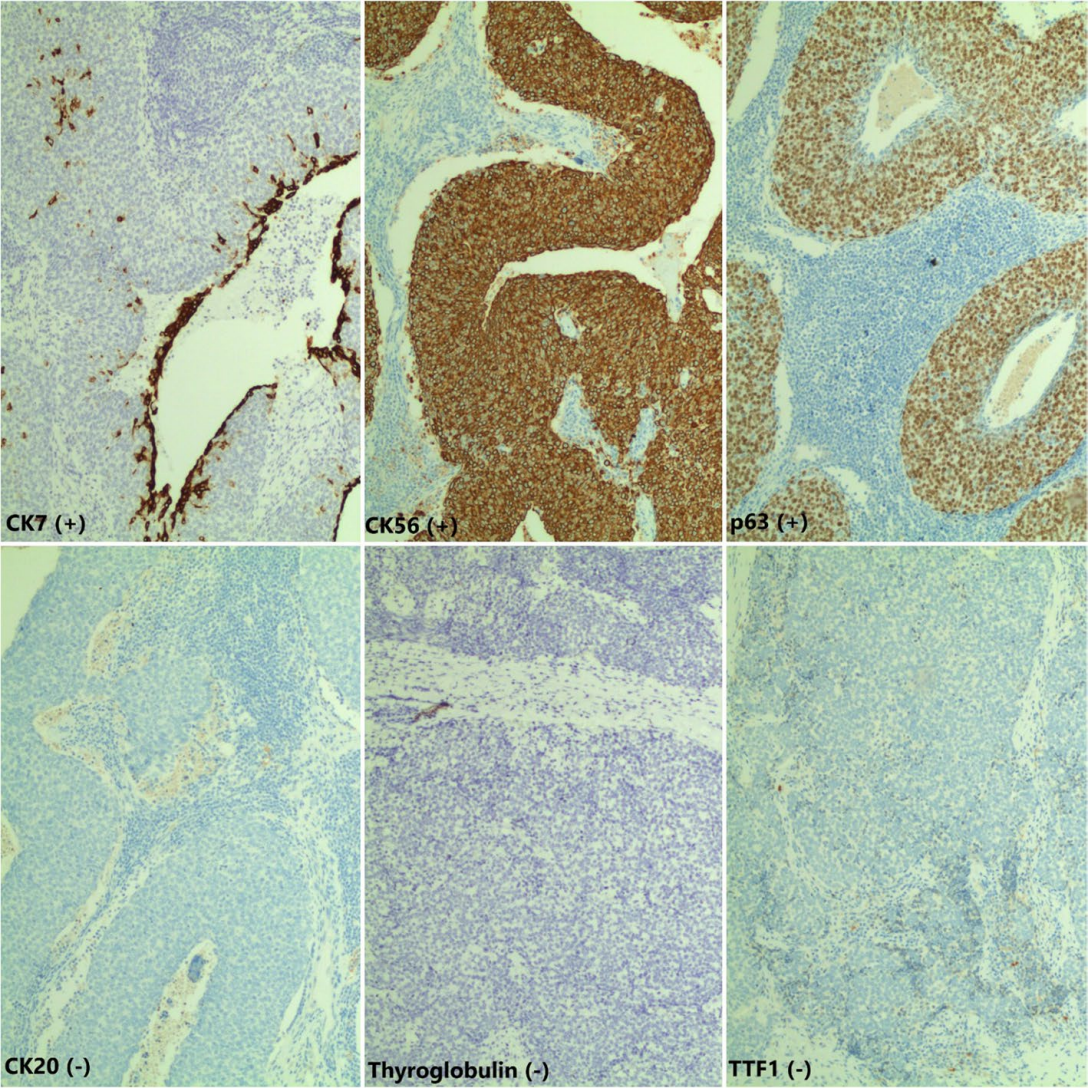
Result of immunohistochemistry: marker CK7, CK56, p63 ( +); marker CK20, Thyroglobulin, TTFI (-)

CASTLE arises in the thyroid gland or the soft tissue in the neck. It is necessary to differentiate CASTLE from the other tumors such as primary or metastatic head and neck squamous cell carcinomas or carcinomas of the thyroid gland because the prognosis and treatment are different. Diagnostic imaging methods include neck ultrasound, CT scan, and MRI. CASTLE tumors on CT scan often show an unclear boundary and no calcification in the lesion. Fine needle aspiration cytology plays an important role in the diagnosis of thyroid cancer. However, cytology cannot differentiate CASTLE from less differentiated thyroid cancer such as squamous carcinoma or undifferentiated thyroid carcinoma [6, 9]. A needle biopsy can obtain a tissue sample with an appropriate size for immunohistochemistry. The IHC analysis of the CASTLE tumor shows a strong positive staining with CD5, p63, and cytokeratin but negative staining with thyroglobulin, TTF1, and calcitonin [7, 10]. Positive staining with CD5 marker helps to differentiate CASTLE from other tumors of the thyroid, respiratory tract, or upper gastrointestinal tract [11]. We found similar results on pathology and IHC analyses in our case: the lesion was positive with CK5, CK7, p63, C-kit, and CD5 but negative with TTF-1, Thyroglobulin, GATA3, ER, CD99, and NKX2.2 (Fig. 3).

Due to the rarity of the disease, there are currently no standard treatment guidelines, but surgery is usually considered the first choice. According to the reports, the rates of extracellular invasion and lymph node metastases are relatively high, at 50–60% and 50% respectively [1, 12, 13]. It has also been reported that patients receiving curative surgery including total thyroidectomy and cervical lymph node dissection had favorable results, with regional recurrence rates of 14%, and 5 years and 10 years survival rates of 90 and 82% respectively [14]. Therefore, complete resection of the tumor, including removal of invaded organs, is essential to reduce rates of local recurrence and improve survival rates. CASTLE is considered a disease that has a good response to radiotherapy [9]. In the case series of Tsutsui H et al. [6], one patient refused surgery and was treated with radiotherapy only; the tumor showed a complete response, and a follow-up for 7 years with CT scans did not show a recurrence. In a series of 10 patients with CASTLE tumor (9 patients with a breaking thyroid tumor) who underwent surgery and adjuvant radiotherapy, only 4 patients had a recurrence, all outside the irradiated areas [10]. It has been suggested that patients with CASTLE should undergo surgery to completely remove tumors followed by adjuvant radiotherapy. Also, some authors have suggested that postoperative radiotherapy should be considered for patients with positive or suspected lymph node involvement. As reported by Roka and Piacentini, surgery appears to be sufficient for patients who do not have lymph node metastases, as any of the patients in their series relapsed [1, 15]. In the study by Tsutsui H et al. [6], 2 patients who had no lymph node metastases and did not receive postoperative radiotherapy, were followed up for 5 years and 10 years respectively without showing recurrence. Sun et al. have shown that it rarely recurred after lymph node removal at initial surgery with negative pathology results [11]. For patients who have a recurrence after the initial treatment, surgery and radiotherapy still play an important role rather than chemotherapy. Some reports have indicated lower rates of local recurrence in the group of patients who had locally invasive tumors and cervical lymph node metastases when treated with radiotherapy [10]. However, in our case, the patient and her family decided not to undergo treatment for the recurrence. At present, after 15 months of follow-up, the patient remains stable, suggesting conservative management is an acceptable approach in selected cases of recurrence when surgery is not feasible.

## Conclusion

CASTLE is a rare disease, which has no specific signs and symptoms. Diagnosis of this disease is based on postoperative pathology and immunohistochemistry analysis, especially upon CD5 marker. Treatment for the disease includes surgery and radiotherapy. Curative surgery including total thyroidectomy and lymphadenectomy helps to reduce the recurrence rate and improve survival. Adjuvant radiotherapy should be used for patients who have lymph node metastases and recurrent tumors that are unsuitable for resection. In selected cases, conservative management with follow-up is also an option for such patients.

## Data Availability

The datasets used during the current study are available from the corresponding author on reasonable request.

## Abbreviations

CASTLE: Carcinoma showing thymus-like differentiation
ENT: Ear, Nose, and Throat
CT: Computed Tomography
PET CT: Positron Emission Tomography—Computed Tomography
MRI: Magnetic resonance imaging
FDG: Fluorodeoxyglucose
SUV: Standardized Uptake Value
IHC: Immunohistochemistry
SETTLE: Spindle epithelial tumor with thymus-like differentiation
